# A Longitudinal Study of the Associations of Family Structure with Physical Activity across the Week in Boys and Girls

**DOI:** 10.3390/ijerph16204050

**Published:** 2019-10-22

**Authors:** Emma Solomon-Moore, Ruth Salway, Lydia G. Emm-Collison, Simon J. Sebire, Janice L. Thompson, Russell Jago

**Affiliations:** 1 Centre for Exercise, Nutrition & Health Sciences, School for Policy Studies, University of Bristol, 8 Priory Road, Bristol BS8 1TZ, UK; E.Solomon-Moore@bath.ac.uk (E.S.-M.); Lydia.Emm-Collison@bristol.ac.uk (L.G.E.-C.); Simon.Sebire@bristol.ac.uk (S.J.S.); Russ.Jago@bristol.ac.uk (R.J.); 2 School of Sport, Exercise and Rehabilitation Sciences, University of Birmingham, Birmingham B15 2TT, UK; J.Thompson.1@bham.ac.uk

**Keywords:** physical activity, children, families, family structure, longitudinal, accelerometry

## Abstract

The aim of this study was to examine how family structure is associated with moderate-to-vigorous-intensity physical activity (MVPA) for children aged between 6 and 11. At 6, 9 and 11 years, children wore an accelerometer and parents/carers completed questionnaires on demographics and family structure. Linear regression models examined cross-sectional associations between family structure and MVPA at age 9 and 11. Linear multilevel models examined longitudinal associations between age 6 and 11, differences in change over time were examined using interaction terms. No associations between exposures and MVPA were evident at age 9. Compared to boys living in one home, eleven-year-old boys who lived in multiple homes performed 15.99 (2.46–29.52) fewer minutes of MVPA on weekend days. In longitudinal analyses, the evidence was unclear whether the association with family structure changed over time. Models that assumed associations with family structure remained constant over time, found that boys who lived in multiple homes performed 11.02 (0.76–21.28) fewer minutes of MVPA per weekend day, while for each additional sibling, girls performed an extra 1.89 (0.25–3.53) minutes of MVPA per weekend day. Findings indicate a small number of associations, varying in magnitude, between family structure and children’s MVPA. Therefore, families of all structures should be supported to help their children meet MVPA recommendations.

## 1. Introduction

Being physically active during childhood reduces risk factors for cardiovascular disease and type 2 diabetes [1], as well as improving emotional well-being and self-esteem [2]. The United Kingdom (UK) Chief Medical Officers recommend that children and adolescents engage in at least 60 minutes of moderate-to-vigorous-intensity physical activity (MVPA) per day [3]. However, nationally-representative data from the UK suggest that only 51% of 7–8-year-old children meet these recommendations [4]. Therefore, to inform the development of strategies to increase children’s physical activity, it is important to understand what factors are associated with physical activity behaviour in children, and how these factors are associated with change in physical activity as children age.

Physical activity declines as children age [5,6,7]. A large multi-national longitudinal study of children and adolescents (aged 2–18 years) found that, after five years of age, there was an average cross-sectional decrease of 4.2% in total physical activity with each additional year of age [5]. Similarly, it is well established that boys are more active than girls at all ages [5,6,7,8] and that girls exhibit larger declines in physical activity than boys [6,9,10]. Families are considered to be an important influence on children’s physical activity [11,12,13,14]. The way families are structured is changing, with the ‘traditional’ two-parent nuclear family becoming less dominant, while single-parent households and multi-family households are becoming more prevalent [15]. For instance, in the UK, there were over 1.7 million single-parent households with dependent children in 2017, and households containing two or more families were the fastest growing household type between 2007 and 2017 [15]. In addition, family size is also reducing, with the average number of dependent children within UK families decreasing from 2.0 in 1970 to 1.75 in 2017 [15]. There is a need to understand how contemporary family structures are associated with children’s physical activity.

Studies examining the association between family structure and children’s physical activity report inconsistent findings. A few cross-sectional studies have indicated that a greater number of siblings and living in a two-parent home are associated with higher levels of physical activity among children, especially for girls [12,13], while others have shown that living in a single-parent household is associated with higher physical activity levels [16,17,18]. In contrast, several studies found no association between family structure and physical activity [19,20,21,22,23]. The few studies examining parental marital status in association with longitudinal change in physical activity have found no evidence for associations among children aged 9 years and younger [24,25] or 10–13 years [26,27].

There is wide variability across studies in the definition of family structure [28], with the majority of studies only examining parental marital status, the number of parents in the household, and/or the number of children/siblings. No studies to date have examined the influence of living across more than one home, or how family structure may be associated with variation in children’s physical activity on weekdays and weekend days. Additionally, the majority of studies used self-reported measures of physical activity [16,19,20,21,23,24,27], and only two examined samples from the UK [23,26]. There is a need to examine cross-sectional associations between physical activity and family structure to ascertain whether the associations present in other datasets are replicated in the UK using objective measures. Additionally, it is important to understand whether any associations persist or are exacerbated as children age. We know that physical activity levels decline as children age [5,6], but the impact that family structure has on those associations is as yet unknown. Therefore, conducting analyses to understand how family structure is associated with physical activity as children age will improve the ability of researchers and policy makers to target family-based physical activity intervention efforts. To address these gaps, the aim of the present study was to investigate the cross-sectional associations between family structure and children’s accelerometer-assessed physical activity at age 9 and 11 years how these associations varied across the week and by child gender, and whether any associations change between ages 6 and 11.

## 2. Materials and Methods

The data used in the present study are from the longitudinal B-PROACT1V study [6,7,29,30], which aimed to examine children’s and parents’ physical activity and sedentary behaviours during primary school. Between February 2012 and July 2013, 250 primary schools, located in Bristol, UK, and the surrounding area, were invited to participate in this study. Sixty-five (26%) schools consented to participate, but two withdrew before any children had been recruited and data collection could not be scheduled in a further six. Therefore, baseline data were collected from the remaining 57 primary schools, with 1299 children taking part when they were in Year 1 of school (median age: 6 years). Between March 2015 and July 2016, 47 of the original schools were re-recruited, and data were collected from 1223 Year 4 children (median age: 9 years). Between March 2017 and July 2018, 50 of the original schools were re-recruited, and data were collected from 1296 Year 6 children (median age: 11 years). All children in the corresponding year groups within each school were eligible to participate in this study at each time point. In total, 2132 children participated, of whom 958 participated at one time point, 662 at two time points, and 512 at three time points. At least one of the children’s parents/carers were also recruited to this study at each time point. Due to limited family structure information being collected at age 6, the current study used quantitative data from the children who provided data at either age 9, age 11 and/or both age 9 and 11 (N = 1714). The sample was limited to participants who provided valid child accelerometer data and parent questionnaire data for questions on family structure. Ethical approval for this study was granted from the School for Policy Studies Research Ethics Committee at the University of Bristol and written parent consent was provided for both parent and child participation.

### 2.1. Accelerometer Data

At each time point, children were asked to wear a waist-worn ActiGraph wGT3X-BT accelerometer for all waking hours on five days, including two weekend days. Accelerometer data were processed using Kinesoft (V.3.3.75; Kinesoft, Saskatchewan, Canada). Accelerometer data were included in the analyses if children provided at least three days of valid data. A day was considered valid if there was at least 500 minutes of data after excluding intervals of ≥60 minutes of zero counts, allowing up to two minutes of interruptions. The mean number of MVPA minutes per day overall and separately by weekdays and weekend days for the children were derived using population-specific cut points for children [31]. For longitudinal models, we used accelerometer data collected at age 6 where available.

### 2.2. Family Structure Variables

Depending on their preference, parents/carers were either sent a paper questionnaire or emailed a link to an online questionnaire. At the age 9 and 11 data collections, parents were asked to report the number of siblings the participating child has, what other adults (over 18 years) live in the home, and whether their child lives in the same house as them all or part of the time (e.g., weekdays/weekends only). Number of siblings was treated as a continuous variable. The question on the other adults that live in the home had the following response options: ‘no-one’, ‘female caregiver (mother/step-mother)’, ‘male caregiver (father/step-father)’ and ‘other (please specify)’. This enabled us to derive a binary variable quantifying how many parents or caregivers live in the household; ‘one’ or ‘two or more’. The response options for how often their child lives in the same house as them were: ‘all of the time’, ‘weekdays only’, ‘weekends only’, ‘some of the time (mix of weekdays/weekends)’, and ‘other’. The vast majority of parents reported that their child lives with them ‘all of the time’ (95.8%). Therefore, a binary variable was derived, indicating how many homes a child lives in: ‘one’ or ‘two or more’. To provide baseline measures of family structure variables at age 6 in the longitudinal analyses, parents were asked at baseline to report the number of siblings the participating child has. Information was not collected at baseline on the ‘number of parents/carers live in the household’ or ‘how many homes a child lives in’. Therefore, these variables were derived from data collected at age 9 and age 11, in combination with information provided by parents on household/family changes between age 6 and age 9.

### 2.3. Potential Confounders

Parents provided children’s demographic information via the questionnaire, including gender and date of birth. Where children’s date of birth was missing (9.5% of children at Year 1, 8.5% of children at Year 4, 8.5% of children at Year 6), they were assigned the median age of 6.0 years at Year 1, 9.0 years at Year 4, and 11.0 years at Year 6. As indicators of socioeconomic status, parents were asked to report the highest level of education in the home, with the following response options: ‘up to GCSEs/ GCEs/ O Levels or equivalent’ (qualifications usually obtained at age 16), ‘A Levels/ NVQs/ GNVQs’ (qualifications usually obtained at age 18), ‘Degree/ Diploma/ HNC/ HND or equivalent’ and ‘Higher degree (MSc, PhD)’. This was combined across time points to produce a single indicator of highest household education. Trained researchers conducted data measurement sessions within the schools, where child height was measured to the nearest 0.1 cm using a SECA Leicester stadiometer (HAB International, Northampton) and weight was measured to the nearest 0.1 kg using a SECA 899 digital scale (HAB International, Northampton). These were used to derive children’s body mass index (BMI) as weight (kg)/ height (m)^2^, and this was converted to an age- and gender-specific standard deviation score [32].

### 2.4. Statistical Analysis

Means and proportions were used to examine the distributions of exposures and outcomes between genders at different time points. Linear regression models were used to examine the cross-sectional associations between the individual family structure exposure variables (number of siblings, number of parents/carers in the household, number of households child resides in) and weekday and weekend MVPA at ages 9 and 11. Partially adjusted models (Model 1) were adjusted for child age, BMI z-score, highest household education, and weekday/weekend accelerometer wear time as potential confounders. In the fully adjusted models (Model 2), we additionally adjusted for the other exposure variables. We analysed MVPA on weekdays and weekend days separately, as previous research [33,34] suggested that levels and patterns of physical activity on these days may differ. As evidence consistently shows that physical activity patterns differ by gender, with girls engaging in less activity than boys, the models were run separately for boys and girls [9,35,36,37]. Robust standard errors were used to account for the clustering of children in schools for all cross-sectional models.

Linear multilevel models, with random effects at the school and child level to account for clustering and repeated measures, respectively, were used to determine associations of family structure variables with mean levels of physical activity at baseline (age 6 years) and with the change in physical activity over time. We assumed a linear change in activity over time between ages 6 and 11, as we only have data at three distinct ages, and used restricted maximum likelihood for more accurate estimation of standard errors. The multilevel models were run with and without an interaction term between age and the exposure variable of interest in order to examine whether the association with family structure changes over time. Multilevel models maximise the use of data as they include all participants who have data for at least one measure. Partially adjusted models (Model 1) were adjusted for child age, BMI z-score, highest household education, and weekday/weekend accelerometer wear time. Fully adjusted models (Model 2) were additionally adjusted for the other exposure variables. All analyses were performed in Stata V.15.1 (StataCorp, 2015).

In total, 1026 (83.9%) children with valid accelerometer data on weekdays (931 (76.1%) at weekend) were included in the age 9 cross-sectional model, and 1062 (81.9%; 940 (72.5%) at weekend) were included in the age 11 cross-sectional model (Table 1). Missing family structure exposure information varied from 16.0% for girls’ number of siblings at age 11 to 23.4% for the number of houses girls live in at age 11. Missing covariate information varied from 0.4% to 8.2%. Complete data were available for a total of 791 participants (64.7%) at age 9 and 745 participants (57.5%) at age 11. The longitudinal analyses were based on 1491 (87.0%) children who had valid weekday accelerometer data for at least one time point (1375 (80.2%) at weekend), with 597 (34.8%) children having valid weekday accelerometer data at both time points (496 (28.9%) at weekend).

## 3. Results

Descriptive characteristics at ages 9 and 11 are summarised in Table 2. Boys were more active than girls at both time points. Weekday MVPA remained static for boys but decreased with age for girls, while weekend MVPA decreased with age for both genders. Average BMI z-score was highest at age 11 for boys and age 9 for girls. Over half the sample lived in households where one parent/carer was educated to University degree level or higher, approximately 85% had at least one sibling and lived in homes with two or more parents/carers, and over 90% of children lived in one home across the week.

There was no cross-sectional evidence of associations between family structure variables with weekday or weekend MVPA at age 9 for either boys or girls (Table 3). The number of homes a child lives in was associated with weekday and weekend MVPA at age 11 for boys (Table 4). Boys who lived across two or more homes performed 9.58 (95% confidence interval: 1.69 to 17.47) fewer minutes of MVPA per weekday compared to similar boys who lived in one home (partially adjusted model only). Similarly, boys who lived across two or more homes performed 15.99 (2.46 to 29.52) fewer minutes of MVPA per weekend day in the fully adjusted model compared to similar boys who lived in one home. In the partially adjusted model, boys with two or more parents/carers at home performed an additional 10.72 (1.46 to 19.99) minutes of MVPA per weekend day compared to similar boys with one parent/carer at home, but this association was not evident in the fully adjusted model.

In the longitudinal models, the interaction models provided no evidence of family structure changing over time (Table 5). In the models where the association with family structure was assumed to be constant over time, the number of homes a child lives in was associated with weekend MVPA for boys, whereby boys who lived across two or more homes performed 11.02 (0.76 to 21.28) fewer minutes of weekend MVPA compared to similar boys who lived in one home (Table 6). The number of siblings a child has was weakly associated with weekend MVPA for girls. For each additional sibling, girls performed an additional 1.89 (0.25 to 3.53) minutes of MVPA per weekend day. There were no associations between family structure variables with weekday MVPA for either boys or girls.

## 4. Discussion

This is the first study to examine cross-sectional and prospective associations of multiple family structure variables with physical activity on weekdays and weekend days during childhood. The findings suggest that the extent to which family structure is associated with physical activity varies by exposure variable, child age and gender. Previous studies have been inconclusive about the association between family structure and physical activity. While a few cross-sectional studies suggest that living in a single-parent household is either negatively [12,13] or positively [16,17,18] associated with children’s physical activity, the majority of studies found no evidence for associations [19,20,21,22,23,24,25,26,27]. In the present study, the number of parents/carers in the home was associated with weekend MVPA for eleven-year-old boys, where boys with two or more parents/carers at home were more physically active than boys from a single-parent household by 11 minutes per weekend day. However, this association was only evident in the partially adjusted model, not in the fully adjusted or longitudinal models.

To our knowledge, this was the first study to examine the association between living across more than one home and child physical activity. In the cross-sectional models, the number of homes that boys lived in was associated with weekday and weekend MVPA at age 11, where boys who lived in more than one home were less physically active than boys who lived in one home by 10 minutes per weekday (in the partially adjusted model, no association in the fully adjusted model) and 16 minutes per weekend day. In order to examine longitudinal associations with family structure, prospective analyses were conducted between baseline (age 6) and 11 years, with and without an interaction term for age. When the association with family structure was assumed to be constant over time, boys who lived across two or more homes did 11 fewer minutes of MVPA per weekend day, compared to similar boys who lived in one home. The interaction models revealed no evidence of the association with family structure changing over time. However, at age 6, boys who lived in more than one home engaged in one minute less of MVPA per weekend day that their counterparts in single homes, with the difference increasing at a rate of three minutes/day/year. Considering the cross-sectional and longitudinal results together, a change over time of approximately 3 minutes/year is perhaps more plausible than a constant effect of 11 minutes, but the low number of boys in the sample that lived across more than one home (<10%) limited our ability to detect changes. It may be that living across more than one home is disruptive to boys’ physical activity at weekends, due to time spent passively travelling between homes or schedule changes that affect time spent on physical activity. Further studies are warranted to examine this association with larger sample sizes of children living in more than one home.

Previous cross-sectional evidence has suggested that having siblings is positively associated with physical activity, especially among girls [12,13]. In support of this, our prospective findings suggest that for each additional sibling, girls performed an extra two minutes of MVPA per weekend day, and that this association was constant over time. It may be that girls without siblings have fewer opportunities for companion play and therefore spend more time in solitary pursuits, which are often sedentary. A recent qualitative study found that parents restrict their children’s independent mobility and outdoor play due to concerns about safety [38], and so it may be that girls without siblings have more restrictions on their outdoor play on weekends. However, the associations between the number of siblings and girls’ physical activity in the present study were only very small. Therefore, caution should be taken when interpreting these results. It may be that sibling age and gender are important influences on children’s physical activity. Therefore, future studies should investigate how having younger versus older, and gender matched versus unmatched siblings are associated with both girls’ and boys’ physical activity.

The findings of the present study suggest that more research needs to be done to understand in-depth the impact of living across multiple homes on children’s physical activity. It may be that researchers and practitioners need to find ways to engage both parents/carers in families that are living in separate homes to provide them with practical support and planning advice to optimise their child’s ability to meet physical activity recommendations both during the week and on weekends. Similarly, the present research suggests that being an only child may be detrimental to girls’ physical activity on weekends. Therefore, future research could explore strategies to overcome this, for instance via a buddy system, where families with similar-aged children from the same neighbourhood are encouraged to allow girls to play together outside, with supervision responsibilities shared between families to help alleviate safety concerns.

### Strengths and Limitations

Strengths of this study include the objective measurement of physical activity (via accelerometers) at three ages in childhood, spanning five years, which allowed us to examine longitudinal associations between family structure exposure variables with change in physical activity between the ages of 6 and 11 years. This study is limited by missing data, especially weekend physical activity and parent-reported family structure variables. Only a small proportion of children in the sample lived in more than one household, which limited the ability to make inferences from the data. Due to the complexity of the problem being explored, it was not possible to control for all factors that may influence the association between family structure and children’s physical activity. For instance, we did not have complete data on the gender of all members of the family structure, and so we were unable to examine the association between different gender patterns within families and child physical activity. The sample is relatively homogenous, primarily of White British origin from a single UK area, which limits the ability to extrapolate to other regions, countries and contexts.

## 5. Conclusions

The results of the present study indicate a small number of associations of differing magnitude between family structure and children’s physical activity. Boys who lived across multiple homes showed a potentially important decrease in activity levels at weekends as they aged, while girls were marginally more active at weekends if they had more siblings. Therefore, further research is needed to examine the associations between family structure variables, especially living across more than one home, and children’s physical activity, to ascertain whether there are families that could benefit from additional support. Currently, families of all structures should be supported to optimise their child’s ability to meet physical activity guidelines.

## Figures and Tables

**Table 1:** Sample sizes and proportion of missing data.

	Boys (N = 813)				Girls (N = 901)			
	Age 9 (N = 556)		Age 11 (N = 616)		Age 9 (N = 667)		Age 11 (N = 680)	
	N	% Missing	N	% Missing	N	% Missing	N	% Missing
Weekday MVPA ^1^	456	18.0%	480	22.1%	570	14.5%	582	14.4%
Weekend MVPA	417	25.0%	429	30.4%	514	22.9%	511	24.9%
Body mass index (z score)	553	0.5%	613	0.5%	664	0.4%	672	1.2%
Highest household education ^2^	513	7.7%	567	8.0%	612	8.2%	624	8.2%
Number of siblings	452	18.7%	513	16.7%	544	18.4%	571	16.0%
No. of parents/carers at home	451	18.9%	507	17.7%	539	19.2%	567	16.6%
No. of homes child lives in	444	20.1%	481	21.9%	532	20.2%	521	23.4%

^1^ Moderate-to-vigorous-intensity physical activity (MVPA) variables based on participants providing three valid days of accelerometer data. ^2^ Household education is based on information from both time points.

**Table 2:** Descriptive statistics of the sample.

	Boys Mean (SD) or %		Girls Mean (SD) or %	
Variable	Age 9	Age 11	Age 9	Age 11
Weekday MVPA	69.23 (23.27)	69.37 (24.38)	56.31 (19.27)	53.78 (19.24)
Weekend MVPA	68.46 (37.05)	61.59 (35.15)	55.55 (25.85)	46.43 (25.65)
Age	9.03 (0.44)	10.95 (0.38)	9.02 (0.43)	10.95 (0.38)
Body mass index (z-score)	0.28 (1.07)	0.37 (1.14)	0.40 (1.07)	0.33 (1.18)
Highest household education:				
Up to General Certificate of Secondary Education (GCSE)/O level	19.3%	20.6%	18.6%	20.2%
A level/National Vocational Qualification	28.7%	25.4%	28.6%	26.9%
Degree/ Higher National Diploma	35.5%	37.6%	36.1%	35.6%
Higher degree (Masters/Doctorate)	16.6%	16.4%	16.7%	17.3%
Number of siblings	1.34 (1.09)	1.34 (0.89)	1.29 (1.04)	1.36 (0.98)
No. of parents/carers at home:				
One	12.2%	17.8%	14.5%	21.4%
Two or more	87.8%	82.3%	85.5%	78.6%
No. of homes child lives in:				
One	95.1%	91.1%	93.2%	93.1%
Two or more	5.0%	8.9%	6.8%	6.9%

**Table 3:** Cross-sectional associations of weekday and weekend MVPA with family structure variables at age 9.

	Weekday MVPA						Weekend MVPA					
	Boys			Girls			Boys			Girls		
Exposure	N	Coeff	95% Confidence Interval	N	Coeff	95% Confidence Interval	N	Coeff	95% Confidence Interval	N	Coeff	95% Confidence Interval
Number of siblings												
Model 1	389	−0.92	−2.92 to 1.08	479	0.60	−1.01 to 2.21	369	1.48	−1.90 to 4.86	445	1.90	−0.34 to 4.14
Model 2	382	−1.30	−3.32 to 0.73	464	0.43	−1.21 to 2.07	363	1.40	−2.01 to 4.81	433	1.97	−0.30 to 4.25
Two or more (vs. one) parents/carers at home												
Model 1	388	5.43	−1.21 to 12.07	474	1.30	−3.40 to 6.00	369	1.49	−9.58 to 12.56	442	−1.37	−7.99 to 5.25
Model 2	382	5.51	−1.48 to 12.51	464	0.71	−4.30 to 5.71	363	−0.75	−12.45 to 10.95	433	−3.43	−10.45 to 3.60
Child lives in two or more (vs. one) homes												
Model 1	383	−6.29	−17.01 to 4.42	470	−3.08	−9.71 to 3.71	363	−5.33	−22.71 to 12.05	437	−6.42	−15.75 to 2.90
Model 2	382	−4.22	−15.34 to 6.90	464	−2.64	−9.62 to 4.34	363	−5.19	−23.43 to 13.05	433	−7.61	−17.43 to 2.22

Model 1 adjusted for age, BMI z-score, highest household education, and accelerometer wear time (weekday and weekend respectively). Model 2 is additionally adjusted for the other exposure variables. All models are adjusted for clustering at the school level.

**Table 4:** Cross-sectional associations of weekday and weekend MVPA with family structure variables at age 11.

	Weekday MVPA						Weekend MVPA					
	Boys			Girls			Boys			Girls		
Exposure	N	Coeff	95% Confidence Interval	N	Coeff	95% Confidence Interval	N	Coeff	95% Confidence Interval	N	Coeff	95% Confidence Interval
Number of siblings												
Model 1	416	1.43	−0.95 to 3.81	495	0.61	−1.00 to 2.23	376	2.06	−2.00 to 6.13	434	1.41	−0.95 to 3.78
Model 2	387	1.24	−1.28 to 3.75	448	0.33	−1.36 to 2.01	349	1.41	−2.93 to 5.75	396	1.40	−0.99 to 3.78
Two or more (vs. one) parents/carers at home												
Model 1	412	3.16	−2.62 to 8.93	491	−1.85	−5.70 to 1.99	372	10.72	1.46 to 19.99	430	1.47	−4.14 to 7.08
Model 2	387	1.36	−5.09 to 7.80	448	−3.70	−7.81 to 0.40	349	6.32	−4.01 to 16.64	396	0.15	−5.79 to 6.09
Child lives in two or more (vs. one) homes												
Model 1	392	−9.58	−17.47 to −1.69	454	0.05	−6.41 to 6.52	354	−19.96	−32.23 to −7.68	402	−2.62	−11.78 to 6.54
Model 2	359	−8.23	−16.80 to 0.35	448	−2.06	−8.85 to 4.74	349	−15.99	−29.52 to −2.46	396	−3.89	−13.49 to 5.71

Model 1 adjusted for age, body mass index (BMI) z-score, highest household education, and accelerometer wear time (weekday and weekend respectively). Model 2 is additionally adjusted for the other exposure variables. All models are adjusted for clustering at the school level.

**Table 5:** Longitudinal associations of weekday MVPA with change in family structure variables between age 6 and 11.

	Weekday MVPA						Weekend MVPA					
	Boys			Girls			Boys			Girls		
Exposure	N	Coeff	95% Confidence Interval	N	Coeff	95% Confidence Interval	N	Coeff	95% Confidence Interval	N	Coeff	95% Confidence Interval
Number of siblings												
Model 1	756	−1.25	−3.72 to 1.22	811	1.13	−1.12 to 3.38	712	0.79	−3.20 to 4.79	754	2.85	−0.38 to 6.07
Interaction with time		0.34	−0.34 to 1.02		−0.16	−0.73 to 0.41		0.11	−1.04 to 1.26		−0.24	−1.07 to 0.59
Model 2	603	−2.72	−5.97 to 0.53	666	0.71	−1.91 to 3.32	573	1.11	−4.31 to 6.53	621	2.45	−1.50 to 6.40
Interaction with time		0.62	−0.22 to 1.47		−0.11	−0.76 to 0.54		−0.05	−1.53 to 1.43		−0.15	−1.14 to 0.83
Two or more (vs. one) parents/carers at home												
Model 1	639	0.58	−6.36 to 7.52	708	1.74	−3.31 to 6.80	608	−0.91	−11.94 to 10.11	663	−0.55	−7.64 to 6.54
Interaction with time		0.61	−1.08 to 2.30		−0.43	−1.65 to 0.78		2.08	−0.74 to 4.91		0.42	−1.37 to 2.20
Model 2	603	3.18	−5.38 to 11.74	666	3.44	−2.70 to 9.57	573	−8.54	−22.18 to 5.11	621	−0.39	−9.07 to 8.29
Interaction with time		−0.14	−2.17 to 1.89		−1.16	−2.61 to 0.29		3.27	−0.13 to 6.66		0.004	−2.13 to 2.14
Child lives in two or more (vs. one) homes												
Model 1	621	−0.43	−10.19 to 9.33	687	0.01	−7.65 to 7.66	591	−1.30	−16.75 to 14.16	644	−4.56	−15.42 to 6.31
Interaction with time		−1.32	−3.57 to 0.94		0.09	−1.83 to 2.01		−3.04	−6.83 to 0.76		0.46	−2.40 to 3.31
Model 2	603	−5.32	−18.30 to 7.65	666	−5.44	−15.27 to 4.40	573	1.11	−20.47 to 22.69	621	−11.03	−24.70 to 2.65
Interaction with time		−0.03	−2.94 to 2.87		1.17	−1.22 to 3.57		−3.20	−8.22 to 1.81		1.73	−1.76 to 5.21

Model 1 adjusted for age, BMI z-score, highest household education, and accelerometer wear time (weekday and weekend respectively). Model 2 is additionally adjusted for the other exposure variables. Interaction terms for the respective family structure exposure variable with time are used to examine whether associations with family structure variables change over time.

**Table 6:** Longitudinal associations of weekday and weekend MVPA with family structure variables between age 6 and 11.

	Weekday MVPA						Weekend MVPA					
	Boys			Girls			Boys			Girls		
Exposure	N	Coeff	95% Confidence Interval	N	Coeff	95% Confidence Interval	N	Coeff	95% Confidence Interval	N	Coeff	95% Confidence Interval
Number of siblings												
Model 1	756	−0.24	−1.68 to 1.20	811	0.57	−0.55 to 1.69	712	1.10	−1.21 to 3.42	754	2.02	0.48 to 3.57
Model 2	603	−0.62	−2.20 to 0.96	666	0.31	−0.87 to 1.49	573	0.96	−1.62 to 3.53	621	1.89	0.25 to 3.53
Two or more (vs. one) parents/carers at home												
Model 1	639	2.59	−1.48 to 6.67	708	0.25	−2.59 to 3.08	608	5.72	−0.65 to 12.09	663	0.82	−3.13 to 4.77
Model 2	603	2.69	−1.92 to 7.30	666	−0.82	−3.87 to 2.23	573	2.57	−4.73 to 9.88	621	−0.38	−4.67 to 3.91
Child lives in two or more (vs. one) homes												
Model 1	621	−4.95	−10.88 to 0.98	687	0.31	−4.17 to 4.78	591	−11.36	−20.36 to −2.36	644	−3.12	−9.18 to 2.94
Model 2	603	−5.44	−12.00 to 1.11	666	−1.24	−6.07 to 3.58	573	−11.02	−21.28 to −0.76	621	−5.09	−11.65 to 1.46

Model 1 adjusted for age, BMI z-score, highest household education, and accelerometer wear time (weekday and weekend respectively). Model 2 is additionally adjusted for the other exposure variables. All models assume that the association with family structure is constant over time.

## References

[1] Strong W.B., Malina R.M., Blimkie C.J.R., Daniels S.R., Dishman R.K., Gutin B., Hergenroeder A.C., Must A., Nixon P.A., Pivarnik J.M. (2005). Evidence based physical activity for school-age youth. J. Pediatr..

[2] Parfitt G., Eston R.G. (2005). The relationship between children’s habitual activity level and psychological well-being. Acta Paediatr..

[3] Department of Health, Physical Activity, Health Improvement and Protection (2011). Start Active, Stay Active: A Report on Physical Activity from the Four Home Countries’ Chief Medical Officers.

[4] Griffiths L.J., Cortina-Borja M., Sera F., Pouliou T., Geraci M., Rich C., Cole T.J., Law C., Joshi H., Ness A.R. (2013). How active are our children? Findings from the Millennium Cohort Study. BMJ Open.

[5] Cooper A.R., Goodman A., Page A.S., Sherar L.B., Esliger D.W., van Sluijs E.M., Andersen L.B., Anderssen S., Cardon G., Davey R. (2015). Objectively measured physical activity and sedentary time in youth: The International children’s accelerometry database (ICAD). Int. J. Behav. Nutr. Phys. Act..

[6] Jago R., Solomon-Moore E., Macdonald-Wallis C., Sebire S.J., Thompson J.L., Lawlor D.A. (2017). Change in children’s physical activity and sedentary time between Year 1 and Year 4 of primary school in the B-PROACT1V cohort. Int. J. Behav. Nutr. Phys. Act..

[7] Jago R., Salway R., Emm-Collison L., Sebire S.J., Thompson J.L., Lawlor D.A. (2019). Association of BMI category with change in children’s physical activity between ages 6 and 11 years: A longitudinal study. Int. J. Obes..

[8] Taylor R.W., Williams S.M., Farmer V.L., Taylor B.J. (2013). Changes in physical activity over time in young children: A longitudinal study using accelerometers. PLoS ONE.

[9] Nader P.R., Bradley R.H., Houts R.M., McRitchie S.L., O’Brien M. (2008). Moderate-to-vigorous physical activity from ages 9 to 15 years. JAMA.

[10] Ball K., Cleland V.J., Timperio A.F., Salmon J., Crawford D.A. (2009). Socioeconomic position and children’s physical activity and sedentary behaviors: Longitudinal findings from the CLAN Study. J. Phys. Act. Health.

[11] Sallis J.F., Prochaska J.J., Taylor W.C. (2000). A review of correlates of physical activity of children and adolescents. Med. Sci. Sports Exerc..

[12] Bagley S., Salmon J., Crawford D. (2006). Family structure and children’s television viewing and physical activity. Med. Sci. Sports Exerc..

[13] Hesketh K., Crawford D., Salmon J. (2006). Children’s television viewing and objectively measured physical activity: Associations with family circumstance. Int. J. Behav. Nutr. Phys. Act..

[14] Edwardson C.L., Gorely T. (2010). Parental influences on different types and intensities of physical activity in youth: A systematic review. Psychol. Sport Exerc..

[15] Office for National Statistics (2017). Families and Households: 2017.

[16] Robinson C.H., Thomas S.P. (2004). The interaction model of client health behaviour as a conceptual guide in the explanation of children’s health behaviors. Public Health Nurs..

[17] Sallis J.F., Taylor W.C., Dowda M., Freedson P.S., Pate R.R. (2002). Correlates of vigorous physical activity for children in grades 1 through 12: Comparing parent-reported and objectively measured physical activity. Pediatr. Exerc. Sci..

[18] Wang L., Qi J. (2016). Association between family structure and physical activity of Chinese adolescents. BioMed Res. Int..

[19] O’Loughlin J., Paradis G., Kishchuk N., Barnett T., Renaud L. (1999). Prevalence and correlates of physical activity behaviors among elementary schoolchildren in multi-ethnic, low income, inner-city neighborhoods in Montreal, Canada. Ann. Epidemiol..

[20] Sallis J.F., Alcaraz J.E., McKenzie T.L., Hovell M.F., Kolody B., Nader P.R. (1992). Parental behaviour in relation to physical activity and fitness in 9-year-old children. Am. J. Dis. Child.

[21] Sallis J.F., Prochaska J.J., Taylor W.C., Hill J.O., Geraci J.C. (1999). Correlates of physical activity in a national sample of girls and boys in grades 4 through 12. Health Psychol..

[22] Freedson P.S., Evenson S. (1991). Familial aggregation in physical activity. Res. Q. Exerc. Sport.

[23] Gorely T., Atkin A.J., Biddle S.J.H., Marshall S.J. (2009). Family circumstance, sedentary behaviour and physical activity in adolescents living in England: Project STIL. Int. J. Behav. Nutr. Phys. Act.

[24] Findlay L.C., Garner R.E., Kohen D.E. (2009). Children’s organized physical activity patterns from childhood to adolescence. J. Phys. Act. Health.

[25] Sallis J.F., Alcaraz J.E., McKenzie T.L., Hovell M.F. (1999). Predictors of change in children’s physical activity over 20 months: Variations by gender and level of adiposity. Am. J. Prev. Med..

[26] Corder K., van Sluijs E.M.F., Ekelund U., Jones A.P., Griffin S.J. (2010). Changes in children’s physical activity over 12 months: Longitudinal results from the SPEEDY study. Pediatrics.

[27] Dishman R.K., Saunders R.P., Motl R.W., Dowda M., Pate R.R. (2009). Self-efficacy moderates the relation between declines in physical activity and perceived social support in high school girls. J. Pediatr. Psychol..

[28] Loprinzi P.D., Cardinal B.J., Loprinzi K.L., Lee H. (2012). Benefits and environmental determinants of physical activity in children and adolescents. Obes. Facts.

[29] Jago R., Sebire S.J., Wood L., Pool L., Zahra J., Thompson J.L., Lawlor D.A. (2014). Associations between objectively assessed child and parental physical activity: A cross-sectional study of families with 5–6 year old children. BMC Public Health.

[30] Jago R., Thompson J.L., Sebire S.J., Wood L., Pool L., Zahra J., Lawlor D.A. (2014). Cross-sectional associations between the screen-time of parents and young children: Differences by parent and child gender and day of the week. Int. J. Behav. Nutr. Phys. Act.

[31] Evenson K.R., Catellier D.J., Gill K., Ondrak K.S., McMurray R.G. (2008). Calibration of two objective measures of physical activity for children. J. Sports Sci..

[32] Cole T.J., Freeman J.V., Preece M.A. (1995). Body mass index reference curves for the UK, 1990. Arch. Dis. Child.

[33] Jago R., Anderson C.B., Baranowski T., Watson K. (2005). Adolescent patterns of physical activity differences by gender, day, and time of day. Am. J. Prev. Med..

[34] Jago R., Salway R., Lawlor D.A., Emm-Collison L., Heron J., Thompson J.L., Sebire S.J. (2018). Profiles of children’s physical activity and sedentary behaviour between age 6 and 9: A latent profile and transition analysis. Int. J. Behav. Nutr. Phys. Act.

[35] Loucaides C.A., Jago R. (2008). Differences in physical activity by gender, weight status and travel mode to school in Cypriot children. Prev. Med..

[36] Jago R., Fox K.R., Page A.S., Brockman R., Thompson J.L. (2010). Physical activity and sedentary behaviour typologies of 10–11 year olds. Int. J. Behav. Nutr. Phys. Act..

[37] Jago R., Page A.S., Cooper A.R. (2012). Friends & physical activity during the transition from primary to secondary school. Med. Sci. Sports Exerc..

[38] Solomon-Moore E., Emm-Collison L.G., Sebire S.J., Toumpakari Z., Thompson J.L., Lawlor D.A., Jago R. (2018). “In my day…”—Parents’ views on children’s physical activity and screen viewing in relation to their own childhood. Int. J. Environ. Res. Public Health.

